# Differential CD147 Functional Epitopes on Distinct Leukocyte Subsets

**DOI:** 10.3389/fimmu.2021.704309

**Published:** 2021-08-04

**Authors:** Supansa Pata, Sirirat Surinkaew, Nuchjira Takheaw, Witida Laopajon, Kantinan Chuensirikulchai, Watchara Kasinrerk

**Affiliations:** ^1^Division of Clinical Immunology, Department of Medical Technology, Faculty of Associated Medical Sciences, Chiang Mai University, Chiang Mai, Thailand; ^2^Biomedical Technology Research Center, National Center for Genetic Engineering and Biotechnology, National Science and Technology Development Agency at the Faculty of Associated Medical Sciences, Chiang Mai University, Chiang Mai, Thailand; ^3^School of Allied Health Sciences, Walailak University, Nakhon Si Thammarat, Thailand

**Keywords:** CD147, T cell regulation, functional epitope, CD147 extracellular domain, cytokine production

## Abstract

CD147, a member of the immunoglobulin (Ig) superfamily, is widely expressed in several cell types. CD147 molecules have multiple cellular functions, such as migration, adhesion, invasion, energy metabolism and T cell activation. In particular, recent studies have demonstrated the potential application of CD147 as an effective therapeutic target for cancer, as well as autoimmune and inflammatory diseases. In this study, we elucidated the functional epitopes on CD147 extracellular domains in T cell regulation using specific monoclonal antibodies (mAbs). Upon T cell activation, the anti-CD147 domain 1 mAbs M6-1E9 and M6-1D4 and the anti-CD147 domain 2 mAb MEM-M6/6 significantly reduced surface expression of CD69 and CD25 and T cell proliferation. To investigate whether functional epitopes of CD147 are differentially expressed on distinct leukocyte subsets, PBMCs, monocyte-depleted PBMCs and purified T cells were activated in the presence of anti-CD147 mAbs. The mAb M6-1E9 inhibited T cell functions *via* activation of CD147 on monocytes with obligatory cell-cell contact. Engagement of the CD147 epitope by the M6-1E9 mAb downregulated CD80 and CD86 expression on monocytes and IL-2, TNF-α, IFN-γ and IL-17 production in T cells. In contrast, the mAb M6-1D4 inhibited T cell function *via* activation of CD147 on T cells by downregulating IL-2, TNF-α and IFN-γ. Herein, we demonstrated that certain epitopes of CD147, expressed on both monocytes and T cells, are involved in the regulation of T cell activation.

## Introduction

CD147, also called M6 antigen, extracellular matrix metalloproteinase (EMMPRIN) or basigin, is a leukocyte surface molecule and a member of the immunoglobulin (Ig) superfamily (1–3). CD147 molecules are expressed at varying levels in multiple cell types, including epithelial cells, endothelial cells and leukocytes (1–4). On the cell surface, CD147 is expressed as 4 isoforms that differ in the quantity of Ig-like domains in their extracellular region generated by differential splicing and differences in transcription initiation sites (1, 2). Basigin-2, the common form of CD147, consists of an extracellular region with two Ig domains, a transmembrane domain and a short cytoplasmic domain (1, 2). The Ig-like domain 1 and domain 2 of CD147 are homologous to the C and V regions of Ig, respectively (5). Several lines of evidence indicate that the multiple functional properties of CD147 reflect multiple interacting partners (1, 2, 6–8). To date, each domain of CD147 has been identified to interact with a wide range of partners, including CD147 itself, integrins, CD98, caveolin, and cyclophilin (1, 2). Interaction between CD147 and its ligands was demonstrated to involve in the matrix metalloproteinases (MMP) production, cell adhesion and regulation of the immune responses.

Significantly, CD147 has been demonstrated to play a role in many different stages of T cell activity, such as T cell development, activation, proliferation, migration and adhesion (1, 2, 6–10). Functional epitopes located on the Ig-like domains of CD147 in the regulation of T cell activation have been elucidated using various monoclonal antibodies (mAbs) against CD147 (7, 8, 11). The effect of anti-CD147 mAbs on T cell activation and the immune response has led to a therapeutic implication for several diseases (1, 2, 12–16). Anti-CD147 antibodies, antagonistic peptides, and siRNA have shown efficacy for the treatment of asthma-mediated lung inflammation, rheumatoid arthritis, adverse myocardial remodeling, malaria and COVID-19 (1, 2, 12–15). The mechanism behind the involvement of CD147 in T cell activation, however, still needs to be defined clearly. In the current study, we investigated the functional epitopes on CD147 extracellular domains in T cell regulation using various clone of CD147 monoclonal antibodies (mAbs).

We generated several anti-CD147 mAbs and demonstrated that the anti-CD147 mAbs clones M6-1D4, M6-2F9 and M6-1E9 recognize different epitopes located on domain 1 of CD147 (7). In addition, the anti-CD147 mAb clone M6-1F3 also recognized different epitopes with inconclusive locations on the CD147 molecule (7). The anti-CD147 mAb clones M6-1D4, M6-2F9 and M6-1F3 induce cell aggregation (8, 11). However, the anti-CD147 mAb clone M6-1E9 was found to regulate T cell activation (7). In this study, all mAbs and CD147 domain 2-specific mAbs were used for functional study of the CD147 molecule. Using this strategy, our current study explored functional epitopes on the extracellular domains of CD147 in the regulation of T cell activation (7). 

## Materials and Methods

### Antibodies, Reagents and Cells

Anti-CD147 mAbs recognizing domain 1 of CD147 molecule clones M6-1D4 (IgM), M6-1E9 (IgG2a), M6-1F3 (IgM) and M6-2F9 (IgM) were produced in our laboratory (7, 8). The anti-CD147 mAb recognizing domain 2 of CD147 molecule clone MEM-M6/6 (IgG1) was purchased from Exbio (Prague, Czech Republic). The isotype-matched control, mouse mAb clone 4G2 (IgG2a) and Hb1a (IgM) were produced in our laboratory. The anti-CD3ϵ mAb clone OKT3 was purchased from Ortho Pharmaceuticals (Raritan, NJ, USA). FITC-labeled anti-CD3 mAb, FITC-labeled anti-CD4 mAb, APC-labeled anti-CD4 mAb and PE-conjugated anti-CD25 mAb were purchased from BD Bioscience (San Jose, CA, USA). PE-conjugated anti-CD69 mAb, PE-conjugated anti-mouse IgG1 isotype control and FITC-conjugated anti-CD86, MHC class I (HLA-ABC) and MHC class II (HLA-DR) mAbs were purchased from ImmunoTools (Friesoythe, Germany). PECy7-conjugated anti-CD3 mAb, PerCP-conjugated anti-CD14 mAb, PE-conjugated anti-CD19 mAb and PE-conjugated anti-human cytokine antibodies (anti-IL-2, IL-6, IL-17, IFN-γ, TNF-α) were purchased from BioLegend (San Diego, CA, USA).

Peripheral blood mononuclear cells (PBMCs) were isolated from healthy donors using Ficoll-Hypaque (IsoPrep) (Robbins Scientific Corporation, Sunnyvale, CA, USA) gradient centrifugation. To prepare monocyte-depleted PBMCs, monocytes were depleted from PBMCs using Percoll (Amersham Biosciences, Uppsala, Sweden) density gradient centrifugation followed by BD FACSMelody™ Cell Sorter using a PE-conjugated anti-CD14 mAb. The obtained monocyte-depleted PBMCs were confirmed by flow cytometry, and <1% CD14^+^ monocyte contamination was observed. CD3^+^ T cells were prepared by negative isolation using a BD FACSMelody™ Cell Sorter using PE-conjugated anti-CD14 mAb, anti-CD19 mAb, and anti-CD56 mAb anti-CD16 mAb. CD14^+^ monocytes were purified from human PBMCs by a magnetic antibody cell sorting (MACS) system using a Pan Monocyte Isolation Kit (Miltenyi Biotec, Bergisch-Gladbach, Germany). The purity of the obtained CD3^+^ T cells and CD14^+^ monocytes was confirmed by flow cytometry, and >98% purity was obtained.

### T Cell Stimulation and Activation Marker Analysis

The anti-CD3ϵ mAb clone OKT3 was precoated in 24-well plates at a concentration of 25 ng/ml at 4°C overnight. PBMCs were plated at 2×10^6^ cells/well and cultured in the presence or absence of 10 μg/ml anti-CD147 mAbs or isotype-matched control mAbs. Cells were incubated for 18 h at 37°C in 5% CO_2_ and 95% humidified air. Activated PBMCs were harvested, fixed with 4% paraformaldehyde and permeabilized with 0.1% saponin in PBS containing 5% FBS and 0.02% NaN_3_. Fixed cells were stained with PerCP-conjugated anti-human CD3 mAb mixed with PE-conjugated anti-CD25 or anti-CD69 mAbs or isotype-matched control mAb for 30 min at 4°C. Expression of all activation markers was evaluated using a BD Accuri™ C6 flow cytometer (BD Biosciences) and was analyzed using FlowJo software.

For the antibody preactivated CD147 assay, THP-1 cells at a density of 5×10^5^ cells/ml were preincubated with 10 μg/ml anti-CD147 mAbs or isotype-matched control mAbs for 30 min at room temperature. After washing out the excess antibody, preactivated THP-1 cells were cocultured with mAb OKT3-activated PBMCs at a ratio of 1:4 for 18 h at 37°C in 5% CO_2_ and 95% humidified air.

### Cell Proliferation Assay

PBMCs, monocyte-depleted PBMCs or purified CD3^+^ T cells at a density of 1x10^7^ cells/ml were labeled with carboxyfluorescein succinimidyl ester (CFSE; Sigma-Aldrich, St. Louis, USA) at a final concentration of 2 µM for 10 min at 37°C. Excess CFSE was quenched with cold 10% FBS in RPMI. Labeled cells were washed 2 times with RPMI and resuspended in RPMI supplemented with 10% FBS. CFSE-labeled PBMCs at a density of 1x10^6^ cells/ml were plated in 96-well plates with or without immobilized 25 ng/ml anti-CD3ϵ mAb. The anti-CD147 mAbs and isotype-matched control mAb (10 μg/ml) were added to each well. Cells were cultured at 37°C in a 5% CO2 incubator for 5 days, and cell proliferation was analyzed using a BD Accuri™ C6 flow cytometer and FlowJo software.

For the neutralization assay, CFSE-labeled PBMCs were added to anti-CD3ϵ mAb immobilized plates in the presence of anti-CD147 mAbs. The recombinant protein CD147-IgG or CD31-IgG (recombinant protein control) was added to each well. After culturing for 5 days, CFSE-labeled cells were harvested, and proliferation was measured using a BD Accuri™ C6 flow cytometer and analyzed using FlowJo software.

For monocyte-depleted PBMCs and purified CD3^+^ T cells, CFSE-labeled cells were activated with a combination of immobilized 25 ng/ml anti-CD3ϵ mAb and 50 ng/ml soluble anti-CD28 mAb. The anti-CD147 mAbs and isotype-matched control mAb (10 μg/ml) were added to each well. Cells were cultured at 37°C in a 5% CO2 incubator for 5 days, and cell proliferation was analyzed using a BD Accuri™ C6 flow cytometer and FlowJo software.

For the cocultivation assay, purified CD3^+^ T cells and CD14^+^ monocytes were added at a 2.5:1 ratio either in a 96-well flat-bottom plate (Corning, NY, USA) coated with anti-CD3ϵ mAb or in separate compartments of a 96-well plate harboring a 0.4 μm porous polycarbonate Transwell membrane (Corning) containing monocytes in the upper chamber and T cells in the lower chamber coated with anti-CD3ϵ mAb and soluble anti-CD28 mAb. Anti-CD147 mAbs clone M6-1E9 or isotype-matched control mAbs were added to activated cells. After culturing for 5 days, CFSE-labeled cells were harvested, and proliferation was measured by flow cytometry.

### Apoptosis

PBMCs (1X10^5^ cells) were stimulated with 25 ng/ml immobilized anti-CD3ϵ mAb in a 96-well plate in combination with 10 μg/ml anti-CD147 mAbs or isotype-matched control mAbs for 18 h or 5 days at 37°C in 5% CO_2_ and 95% humidified air. Activated cells were harvested and transferred to 96-well V-bottom plates. Cells were washed twice and resuspended in annexin-binding buffer containing 10 mM HEPES/NaOH (pH 7.4), 140 mM NaCl and 2.5 mM CaCl_2_. Annexin V-FITC (Immunotool) and PI (BioLegend) were added to the cell suspension and incubated for 15 min at room temperature in the dark. After the incubation period, 100 μl of annexin-binding buffer was added, and cells were analyzed by flow cytometry within 30 min.

### Costimulatory Molecule Expression Analysis

PBMCs at a density of 1.25×10^6^ cells/mL were stimulated with 25 ng/ml immobilized OKT3 in a 24-well plate in combination with 10 μg/ml anti-CD147 mAb clone M6-1E9 or isotype-matched control mAb. Cells were incubated for 18 h at 37°C in 5% CO_2_ and 95% humidified air. Harvested cells were stained with PE-conjugated anti-CD19 mAb, PerCP-conjugated anti-CD14 mAb or PerCP-conjugated anti-CD14 mAb together with FITC-conjugated anti-CD86, anti-HLA-ABC, HLA-DR (Immunotool) or IgG isotype-matched control mAbs for 30 min at 4°C. Expression of cell surface receptors was evaluated using a flow cytometer and analyzed using FlowJo software.

### Intracellular Cytokine Analysis

PBMCs (2×10^6^ cells) were plated with or without 25 ng/ml anti-CD3ϵ mAb clone OKT3 and soluble anti-CD28 mAb (50 ng/ml) in the presence or absence of anti-CD147 mAbs or isotype-matched control mAb (10 µg/ml) in 24-well plates. After incubation for 1 h at 37°C in 5% CO_2_ and 95% humidified air, protein transport inhibitors, 1 μg/ml brefeldin A and 1 μM monensin, were added and continuously incubated for 5 h at 37°C in a 5% CO_2_ incubator. Activated PBMCs were harvested, fixed in 4% paraformaldehyde and permeabilized with 0.1% saponin in PBS containing 5% FBS and 0.02% NaN_3_. Fixed cells were stained with PECy7-conjugated anti-CD3 mAb, FITC-conjugated anti-CD4 mAb, APC-conjugated anti-CD8 mAb and PE-conjugated anti-cytokine antibody (IFN-γ, IL-2, IL-6, IL-17 or TNF-α) or isotype-matched control mAb for 30 min at 4°C. Intracellular cytokines were quantified by a flow cytometer and analyzed using FlowJo software.

### Statistical Analyses

Data were analyzed using GraphPad Prism 9 software. A two-tailed unpaired t-test was used to compare the means between two groups. Two-way ANOVA with Tukey’s or Sidak’s multiple comparisons posttest was used when the means of more than two groups were compared.

### Human Ethics

This study was approved by the Ethics Committee, Faculty of Associated Medical Sciences, Chiang Mai University, Thailand (AMSEC-60EX-022).

## Results

### Different Functional Epitopes of the CD147 Molecule Regulate T Cell Activation

**Figure 1:**
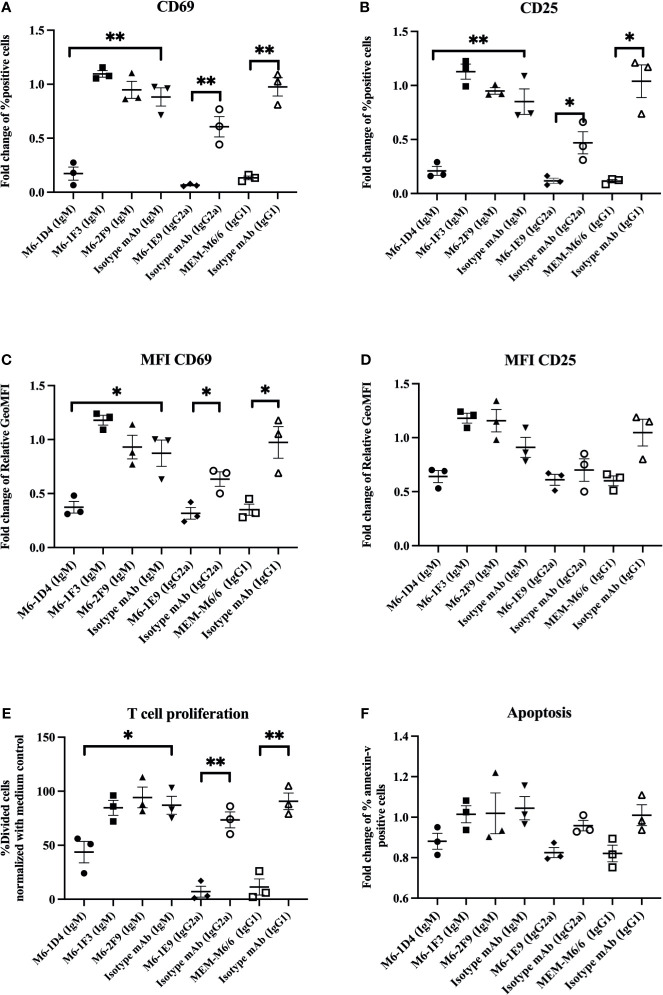
Inhibitory effect of anti-CD147 mAbs on T cell activation. PBMCs (*n=3*) were stimulated with a suboptimal dose of anti-CD3 mAb in the presence or absence of the indicated anti-CD147 mAbs. T cells were gated for the determination of CD69 and CD25 expression. The fold changes of % CD3^+^CD69^+^ cells **(A)** and CD3^+^CD25^+^ cells **(B)** are shown in the graph and indicate the mean ± SE. The fold change of geometric mean fluorescence intensity (GeoMFI) of CD69 **(C)** and CD25 **(D)** are exhibited. The % divided cells (mean ± SE) at day 5 are shown in bar graphs **(E)**. The bar graphs show the fold change of annexin-V-positive cells **(F)**. Fold changes are relative to the medium control condition. Statistical analysis was performed using an unpaired t-test. * represents p ≤ 0.05 and **p ≤ 0.01.

To reveal the epitopes on CD147 that are involved in the regulation of T cell activation, we investigated the effect of different anti-CD147 mAbs on the expression of activation-associated markers, CD69 and CD25, of activated T cells. Results showed that upon T cell activation, the anti-CD147 mAbs M6-1E9, M6-1D4 (react to CD147 domain 1) and MEM-M6/6 (react to CD147 domain 2) significantly reduced CD69 and CD25 expression compared to control conditions (**Figures 1A–D**). The representative FACS profiles were shown in **Figures S1A, B**. With respect to CD69 expression, both the percent of positive cells (**Figures 1A**, **S1A**) and mean fluorescence intensity (MFI) (**Figures 1C**, **S1A**) were significantly reduced. With respect to CD25 expression, the percentage of positive cells (**Figures 1B**, **S1B**), but not the MFI (**Figures 1D**, **S1B**), was significantly reduced. In contrast, the anti-CD147 mAbs M6-1F3 and M6-2F9 (react to CD147 domain 1) did not affect these T cell activation markers.

We next confirmed a previous report on the effect of anti-CD147 mAbs on T cell proliferation. As previously described (7), the CD147 mAbs M6-1E9, M6-1D4 and MEM-M6/6, but not M6-1F3 or M6-2F9, significantly inhibited T cell proliferation (**Figures 1E**, **S1C**). However, we investigated whether the inhibition of cell proliferation by anti-CD147 mAbs is due to induction of apoptosis. We found that among all tested anti-CD147 mAbs, none of them induced apoptosis (**Figures 1F**, **S1D**).

Taken together, we demonstrated that certain epitopes of CD147 domains 1 and 2 play a functional role in the regulation of T cell activation.

### Inhibition of T Cell Proliferation by the Anti-CD147 mAbs M6-1E9, M6-1D4 and MEM-M6/6 Is Due to Their Reactivity to Its Epitope

**Figure 2:**
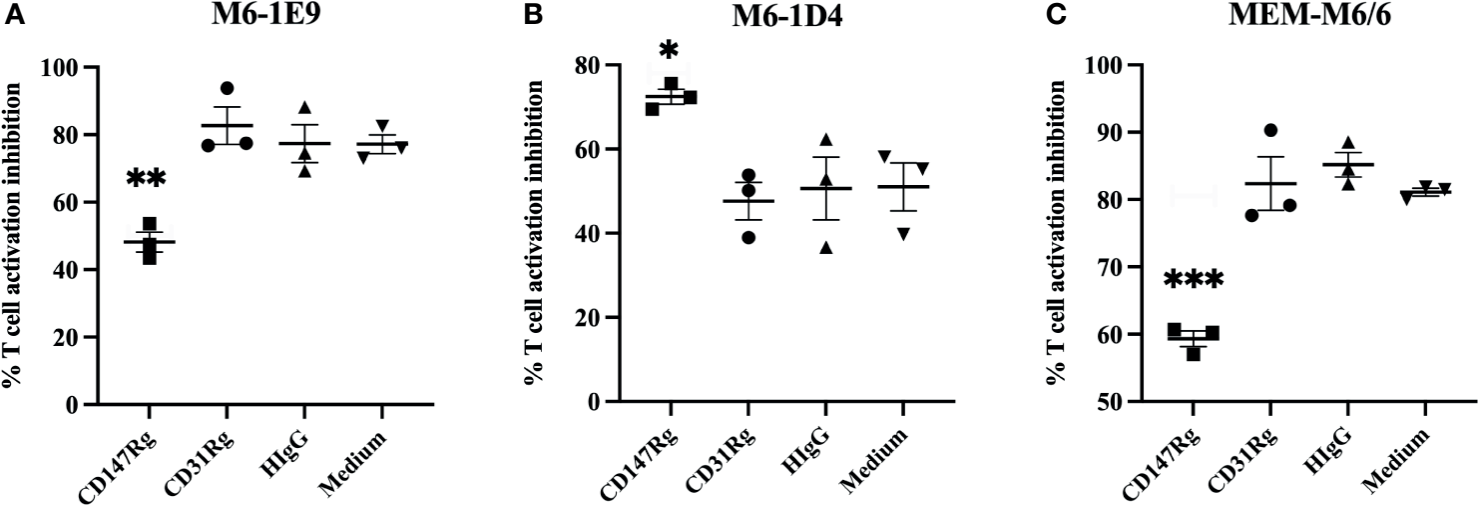
Abolishing of the inhibitory effect of anti-CD147 mAbs through the recombinant CD147 extracellular domain. CFSE-labeled PBMCs (*n* = 3) were stimulated with an anti-CD3 mAb in the presence or absence of an anti-CD147 mAb, **(A)** M6-1E9, **(B)** M6-1D4 or **(C)** MEM-M6/6, mixture with recombinant CD147 proteins as indicated. After 5 days of culture, cell proliferation was determined by flow cytometry. The % proliferation inhibition in the indicated conditions was calculated by normalizing relative to the condition lacking anti-CD147 mAb. The graphs represent the mean ± SE, and two-way ANOVA followed by Tukey’s test was used for comparison. * represents p ≤ 0.05, ** p ≤ 0.01 and ***p < 0.005.

As the anti-CD147 mAbs M6-1E9, M6-1D4 and MEM-M6/6 inhibited T cell proliferation, to confirm that the inhibitory effect was caused by binding of the mAbs to its epitope on the CD147 molecule, a neutralization assay was performed using the recombinant CD147 molecule CD147-IgG (10, 17). The binding ability of mAbs M6-1E9, M6-1D4 and MEM-M6/6 to CD147-IgG was first determined by indirect ELISA and found that all mAbs reacted to CD147-IgG (data not shown). The presence of CD147-IgG, but not CD31-IgG control or human IgG control, diminished the inhibitory effect of the mAbs M6-1E9 and MEM M6/6 (**Figures 2A, B**, **S2**). These findings illustrate that the inhibitory effect of mAbs M6-1E9 and MEM M6/6 is due to binding of the mAbs to its epitope of CD147 molecules. Unexpectedly, a synergistic inhibitory effect of CD147-IgG and the anti-CD147 mAb M6-1D4 was observed (**Figures 2C**, **S2**).

### CD147 Expressed on T Cells and Monocytes Is Involved in the Regulation of T Cell Activation

**Figure 3:**
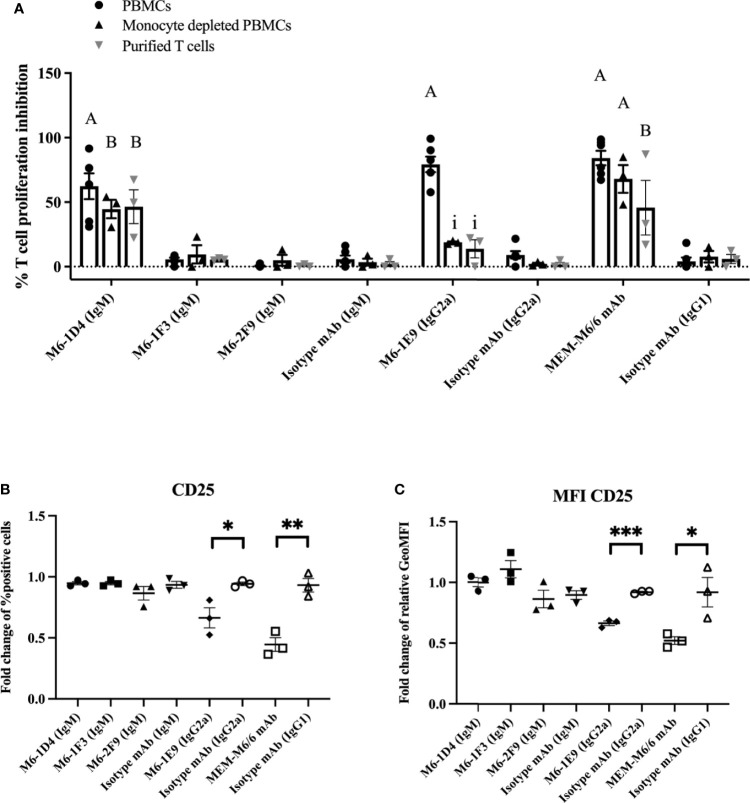
Activation of CD147 on monocytes and T cells with anti-CD147 mAbs regulates T cell activation. PBMCs, monocyte-depleted PBMCs and purified T cells were activated with anti-CD3 mAb or anti-CD3 and CD28 mAbs in the presence of induced anti-CD147 mAbs or isotype-matched control mAbs. The percent proliferation inhibition in the indicated conditions was calculated by normalizing relative to cells lacking anti-CD147 mAb **(A)**. PBMCs were cocultured with the indicated anti-CD147 mAb to preactivate THP-1 cells and activated with an anti-CD3 mAb. The fold change of percent CD25-positive cells in T cells **(B)** and fold change of geometric mean fluorescence intensity (GeoMFI) **(C)** are shown in the graph and indicate the mean ± SE. Fold changes are relative to the medium control condition of the three-five individual donors. Statistical analysis was performed using an unpaired t-test. “A” represents p ≤ 0.0001 and “B” p ≤ 0.01 compared to isotype-matched controls. “i” represents p ≤ 0.0001 compared to PBMCs. * represents p ≤ 0.05 and ** p ≤ 0. 01 compared to isotype-matched controls, ***P < 0.0005.

To investigate which CD147-expressing cell types are key regulators of T cell activation, PBMCs, monocyte-depleted PBMCs or purified CD3^+^ T cells were activated using an anti-CD3 mAb in the presence of anti-CD147 mAbs. As shown in **Figures 3A** and **S3A–C**, in the presence of mAb M6-1D4 or MEM-M6/6, T cell proliferation was significantly inhibited compared to isotype-matched control mAb in cultures of PBMCs, monocyte-depleted PBMCs, and purified T cells. These results indicate that impairment of T cell activation by the mAbs M6-1D4 and MEM-M6/6 is mediated through CD147 domain 1 and domain 2 on T cells, respectively. In contrast, mAbs M6-1F3 and M6-2F9 had no effect on T cell proliferation.

The anti-CD147 mAb M6-1E9, however, significantly inhibited T cell proliferation only in PBMC cultures but not in monocyte-depleted PBMCs or purified T cells. This finding suggests that diminishing T cell activation by the M6-1E9 mAb is mediated through CD147 domain 1 on monocytes. To confirm these observations, THP-1 cells (a human monocytic cell line) were employed in this study. As shown in **Figures 3B, C** and **S3D**, the mAb M6-1E9-preactivated THP-1 cells decreased expression levels of CD25 on activated T cells. The mAb M6-1D4-preactivated THP-1 cells did not affect T cell activation. Surprisingly, THP-1 cells preactivated with the anti-CD147 domain 2 mAb MEM-M6/6 also exhibited decreased expression levels of CD25. These results indicated that the CD147 functional epitopes are located on both extracellular domains and that activation of the CD147 functional epitope is cell type-dependent. It is noted that, by immunofluorescence staining, we confirmed that the mAbs M6-1E9, M6-1D4 and MEM-M6/6 reacted CD147 expressed on surface of monocytes and lymphocytes (**Figure S4**).

**Figure 4:**
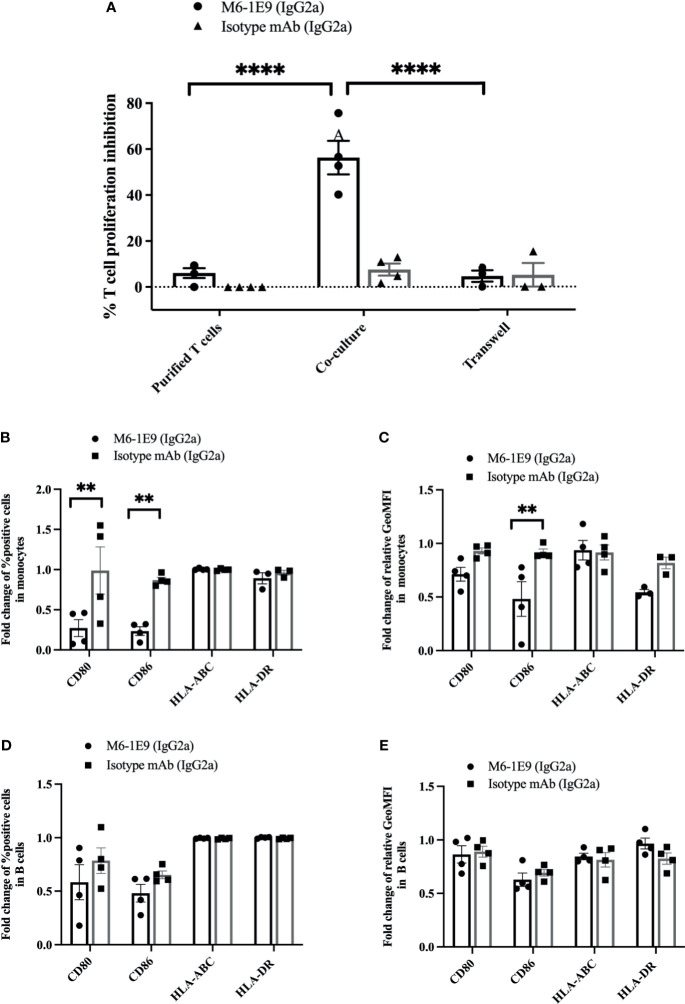
Activation of CD147 on monocytes by the anti-CD147 mAb M6-1E9 regulates T cell activation. Purified T cells and purified T cells cocultured with autologous purified monocytes were activated with anti-CD3 mAb in the absence or presence of mAb M6-1E9 or isotype-matched control mAb. The percent divided T cells under the indicated conditions is shown (*n* = 4). **(A)**. Two-way ANOVA followed by Tukey’s test was used for comparison. PBMCs were activated with anti-CD3 mAb in the absence or presence of mAb M6-1E9 or isotype-matched control mAb. The fold changes of percent CD80-, CD86-, MHC class I (HLA-ABC)-, and MHC class II (HLA-DR)-positive cells on CD14^+^ monocytes **(B)** and CD19^+^ B cells **(D)** are shown in the graph and indicate the mean ± SE. Surface expression levels of CD80, CD86, MHC class I (HLA-ABC), and MHC class II (HLA-DR) on CD14^+^ monocytes **(C)** and CD19^+^ B cells **(E)** were normalized to the medium control. An unpaired t-test was used for comparison, and ** represents p ≤ 0.01 and ****P < 0.0001.

To clarify whether the diminishing T cell activation by mAb M6-1E9-activated monocytes involves a cell-cell interaction or soluble factor secretion, we cocultured T cells and autologous monocytes either together in a 96-well plate or in separate compartments of a 96-well Transwell plate. As shown in **Figures 4A** and **S5A**, under coculture conditions, engagement of CD147 on monocytes by the mAb M6-1E9 inhibited T cell proliferation. In contrast, coculture in separate compartments abolished the mAb M6-1E9 inhibition effect. These results suggest that inhibition of T cell activation *via* engagement of CD147 on monocytes by the mAb M6-1E9 is cell-cell contact-dependent.

We further investigated the effect of mAb M6-1E9 on the expression of costimulatory molecules (CD80, CD86, HLA ABC and HLA-DR) on monocytes and B cells. Activation of CD147 by the mAb M6-1E9 downregulates CD80 and CD86 on monocytes but not B cells (**Figures 4B, C**, **S5B, C**). Expression of HLA-ABC and HLA-DR, however, was not altered (**Figures 4D, E**, **S5B, C**). These findings suggest that activation of the CD147 molecule *via* Ig-like domain 1 by the mAb M6-1E9 downregulates CD80 and CD86 on monocytes, which may subsequently negatively regulate T cell activation.

### CD147 Regulates Cytokine Production in T Cells

**Figure 5:**
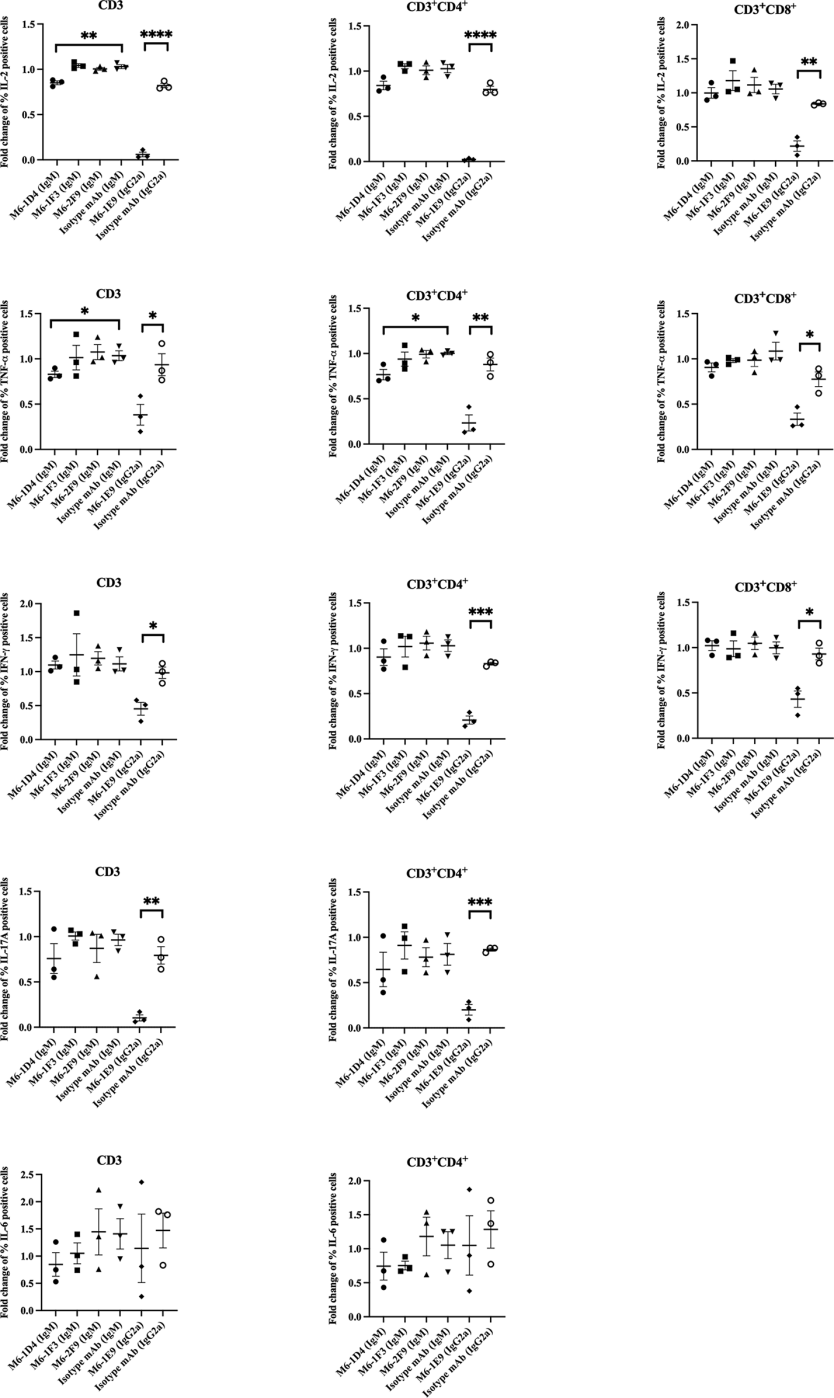
Engagement of CD147 by anti-CD147 mAbsM6-1E9 and M6-1D4 inhibits cytokine production in T cells. PBMCs (*n* = 3) were stimulated with anti-CD3 mAb in the absence (medium control) or presence of the indicated anti-CD147 mAbs or isotype-matched control mAbs. The frequency of cytokine-expressing cells (CD3, CD3^+^CD4^+^ and CD3^+^CD8^+^ T cells) was determined by flow cytometry and normalized to the medium control. Unpaired t-test was used for comparison. * represents p ≤ 0.05 and **p ≤ 0.01, ***P < 0.0005, ****P < 0.0001.

We further investigated the effect of anti-CD147 mAbs on cytokine production. As shown in **Figures 5** and **S6**, upon T cell activation, percentages of IL-2-, TNF-α-, and IFN-γ-producing CD3^+^CD4^+^ and CD3^+^CD8^+^ T cells were significantly decreased in response to the mAbs M6-1E9 and M6-1D4. In addition, percentages of IL-17-producing CD3^+^CD4^+^ T cells were significantly decreased by the mAb M6-1E9. In contrast, mAbs M6-1E9 and M6-1D4 did not affect IL-6 production. The anti-CD147 mAbs M6-1F3 and M6-2F9 had no effect on the production of any of the tested cytokines.

## Discussion

CD147 has been described as a multifunctional molecule in the regulation of immune responses, which is the result of the interaction of its functional epitopes with different ligands (1–4). CD147’s Ig-like domain 1 has been demonstrated to interact with integrin CD98, which is important for MMP induction (1, 2). Ig-like domain 2 has been described to bind with cyclophilins important in the regulation of inflammatory responses and caveolin-1, which is also linked to MMP induction and tumor invasion (1, 2). The transmembrane domain of CD147 binding to CD43, syndecan-1 and β1 integrins was demonstrated to contribute to leucocyte adhesion (1, 2, 18). Both the transmembrane and cytoplasmic domains of CD147 interact with and regulate the transport of monocarboxylate transporters (1, 2). Therefore, anti-CD147 antibodies, acting as agonists, mimicking ligand engagement, or antagonists as blocking agents, have been suggested as potential immunoregulatory biologics.

Upon TCR-mediated activation, administration of the anti-CD147 clone MEM-M6/6, which recognizes Ig-like domain 2, reduced expression of the T cell activation marker CD25 and hence reduced T cell proliferation (10, 19). Transcription of IFN-γ, IL-15, IL-4, and IL-10, but not IL-2, was also diminished (10, 19). Anti-CD147 mAbs that recognize Ig-like domain 1, however, exerted similar and contrasting effects to those of MEM-M6/6 (10, 19). The different results in response to anti-CD147 mAbs might be a result of the different functional epitopes of CD147 that the antibodies recognize. Therefore, we investigated the role of CD147 epitopes located on CD147 extracellular domains in T cell regulation using various clones of anti-CD147 mAbs.

In the current study, the functional epitopes of CD147 in the regulation of T cell activation were identified. Upon T cell activation, the anti-CD147 mAbs M6-1E9 and M6-1D4, which recognize epitopes on CD147 domain 1, significantly reduced surface expression of CD69 and CD25, as well as attenuating T cell proliferation. These results were in accordance with the mAb MEM-M6/6, which reacts to CD147 domain 2 (10, 19). Different epitopes on domains 1 and 2 of CD147 were therefore suggested to play a role in T cell activation. These results are in agreement with a previous study using mAb 5A12 (20). Previous results implied that CD147 on antigen-presenting cells and T cells has important implications for T cell activation (21, 22). Herein, we addressed whether CD147 function is also dependent on the type of cells that are expressed. Focusing on CD147 domain 1, the current study demonstrated that the mAb M6-1E9 significantly inhibited T cell activation only in the presence of monocytes. Moreover, an inhibitory signal appeared when mAb M6-1E9-preactivated monocytic cells were present in the culture. Our findings indicate that the CD147 functional epitope recognized by mAb M6-1E9 exerts its function in monocytes rather than T cells, B cells and NK cells. These results, however, are inconsistent with our previous study demonstrating that inhibition of T cell proliferation with the mAb M6-1E9 did not involve monocytes or CD8^+^ T cells (7). These differences can be explained by the cell preparation techniques being different between the previous and current reports. In a previous report, cell adhesion was used to deplete monocytes from PBMCs, but in the current report, a high-efficiency cell sorter was used. The number of remaining monocytes in monocyte-depleted PBMCs might be the cause of these inconsistent results.

On monocytes, engagement of functional epitopes recognized by the mAb M6-1E9 downregulated CD80 and CD86 expression. CD80 and CD86 are ligands of CD28 on T cells that are necessary for T cell activation (21, 22). Downregulation of CD80 and CD86 expression might interrupt the CD28-CD80/CD86 costimulatory pathway, resulting in inhibition of T cell activation. Accordingly, the effect of mAbs or ligands that downregulate costimulatory molecules on monocytes, leading to inhibition of T cell activation, has been previously reported (23–26).

The functional epitope recognized by the anti-CD147 mAb clone M6-1D4, however, exerts its function differently in T cell activation. This mAb directly triggers its epitope on CD147 in T cells and blocks T cell activation. The signaling pathways generated by this mAb that are responsible for the negative regulation of T cell activation are of interest for future investigation. In addition, by engaging the mAb MEM-M6/6, which reacts to CD147 Ig-like domain 2 (10, 17), our results revealed that the functional epitope recognized by this mAb regulates T cell activation *via* CD147 expression on T cells and monocytes.

Activated T cells secrete various cytokines that mediate immune responses, as well as several autoimmune and inflammatory diseases (27–29). Therefore, we investigated the effect of our mAbs against CD147 domain 1 on cytokine production in various T cell subsets. mAb M6-1E9 reduces IL-2 (7), TNF-α, and IFN-γ in both CD4^+^ T cells and CD8^+^ T cells and IL-17 production in CD4^+^ T cells. However, the mAb M6-1D4 reduces IL-2 (7), TNF-α and IFN-γ in both CD4^+^ T cells and CD8^+^ T cells. For clinical applications, these observations might provide information for strategies designed specifically to suppress the function of activated T cells.

In conclusion, we identified the functional epitopes of CD147, especially Ig-like domain 1, which appears to play a role in T cell activation. In the case of the epitope recognized by mAb M6-1E9, we reached the following conclusions:1) this epitope is a preferential T cell inhibitory factor *via* monocytes but not T cells, B cells or NK cells; 2) activation of this epitope on monocytes results in downregulation of CD80 and CD86 expression and interferes with the costimulatory pathway; 3) this epitope is involved in the regulation of T cell activation, and IL-2, TNF-α, IFN-γ and IL-17 production. For mAb M6-1D4, its recognized epitope is expressed on T cells and plays a role in the inhibition of T cell activation and cytokine production, including IL-2, TNF-α and IFN-γ; in both CD4^+^ T cells and CD8^+^ T cells. The precise molecular mechanism whereby each functional epitope exerts its effect remains to be elucidated in future studies.

This discovery not only lays a solid foundation for an understanding of CD147 in T cell regulation but also provides a potential target of CD147 in immunomodulation for autoimmune and inflammatory diseases.

## Data Availability Statement

The original contributions presented in the study are included in the article/**Supplementary Material**. Further inquiries can be directed to the corresponding author.

## Ethics Statement

This study was approved by the Ethics Committee, Faculty of Associated Medical Sciences, Chiang Mai University, Thailand (AMSEC-60EX-022). Written informed consent for participation was not required for this study in accordance with the national legislation and the institutional requirements.

## Author Contributions

SP provided a conceptual framework for the project, designed and performed experiments, performed data analysis and drafted the original manuscript. SS designed and performed experiments, data analysis and drafted the original manuscript. NT and WL provided guidance for methodology, performed experiments and data interpretation. KC performed experiments and data analysis. WK, the principal investigator, provided guidance for methodology and interpretation of the data, reviewed the manuscript, and preformed editing and finalizing of the manuscript. All authors contributed to the article and approved the submitted version.

## Funding

This work was supported by the Distinguished Research Professor Grant (NRCT808/2563) and the Royal Golden Jubilee PhD program (PHD/0111/2561) of the National Research Council of Thailand. This research project was also supported by TSRI and Chiang Mai University Center of Excellence Project.

## Conflict of Interest

The authors declare that the research was conducted in the absence of any commercial or financial relationships that could be construed as a potential conflict of interest.

## Publisher’s Note

All claims expressed in this article are solely those of the authors and do not necessarily represent those of their affiliated organizations, or those of the publisher, the editors and the reviewers. Any product that may be evaluated in this article, or claim that may be made by its manufacturer, is not guaranteed or endorsed by the publisher.
