# Supplementary material for: Differential CD147 Functional Epitopes on Distinct Leukocyte Subsets
Source: Front Immunol. 2021 Aug 4;12:704309. doi: 10.3389/fimmu.2021.704309 (PMC8371324; doi:10.3389/fimmu.2021.704309)
Supplement: Supplementary file 1 [file DataSheet_1.docx]

**Supplementary Figure S1**


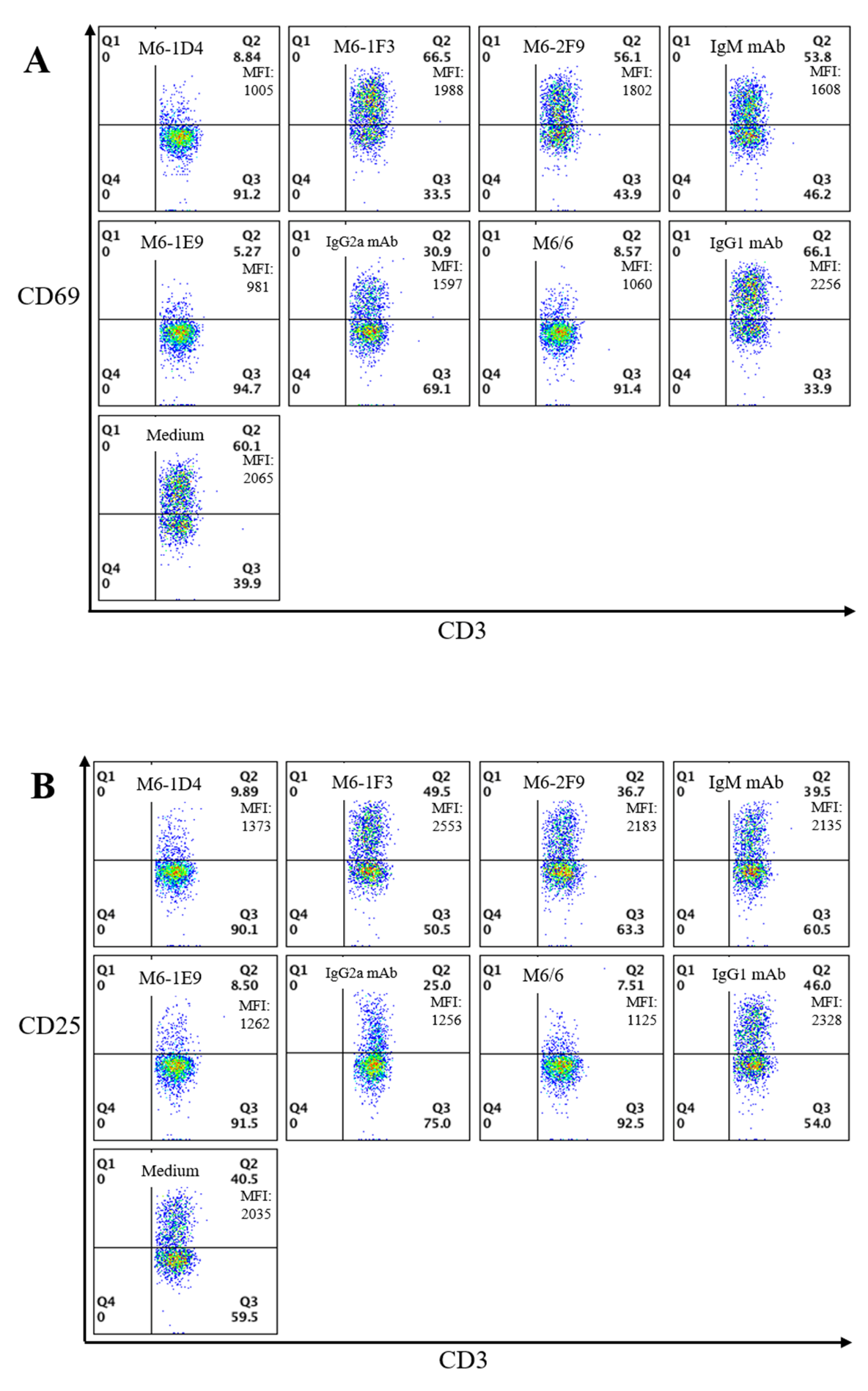


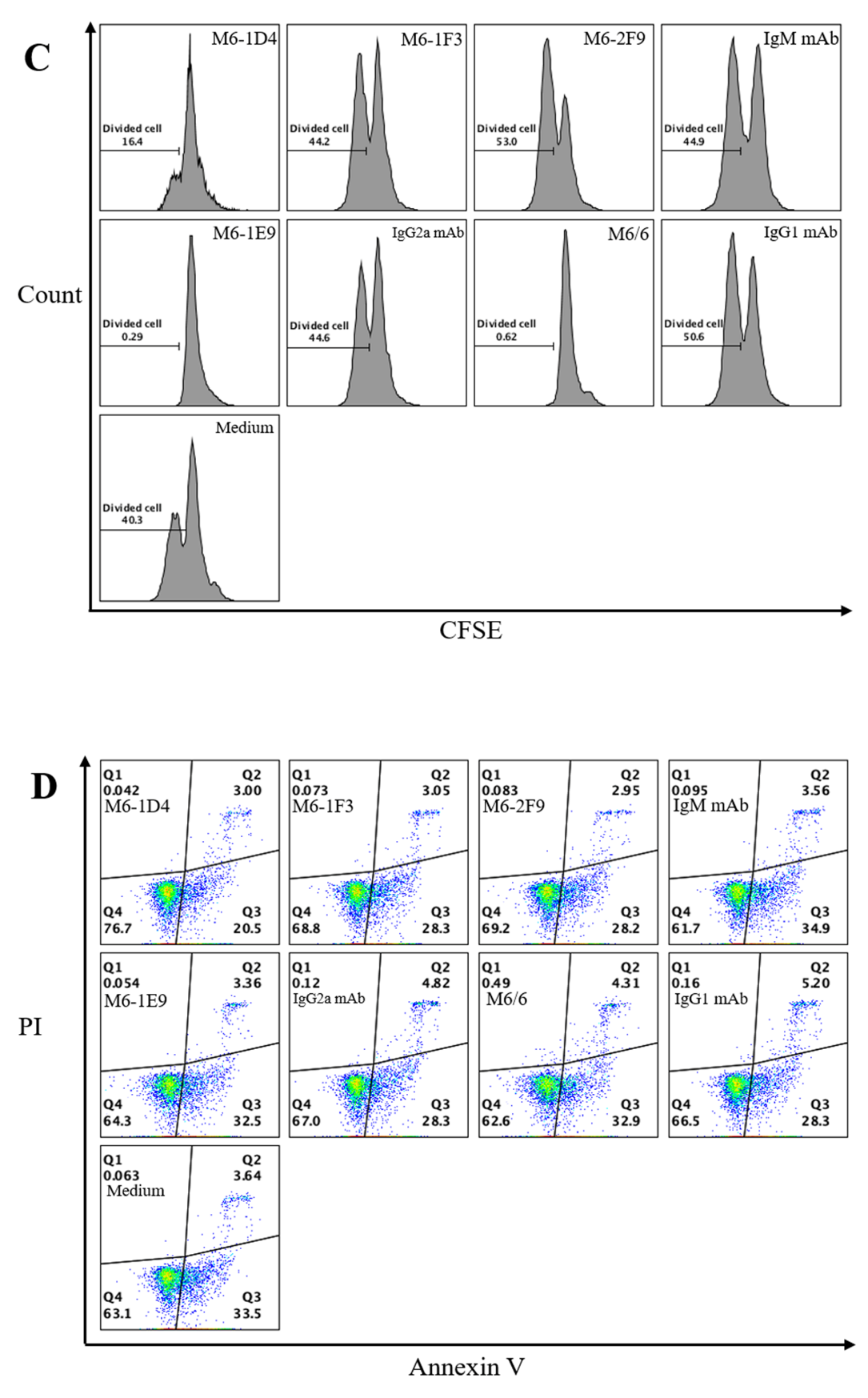


**Figure S1. Inhibitory effect of anti-CD147 mAbs on T cell activation.** PBMCs were activated with anti-CD3 mAb (OKT3) in the absence or presence of indicated anti-CD147 mAbs or isotype-matched control mAbs. Flow cytometric data was expressed in dot plot showing the percentage and geometric mean fluorescence intensity (MFI) of the CD69 **(A)** and CD25 **(B)** expressing T cells in the indicated conditions. **(C)** Flow cytometric data were expressed in histograms showing the percentage of divided cells in each condition using CFSE proliferation assay. **(D)** Flow cytometric analysis shows the percentage of cell apoptosis by Annexin V and PI staining in the indicated conditions.

**Supplementary Figure S2**

**
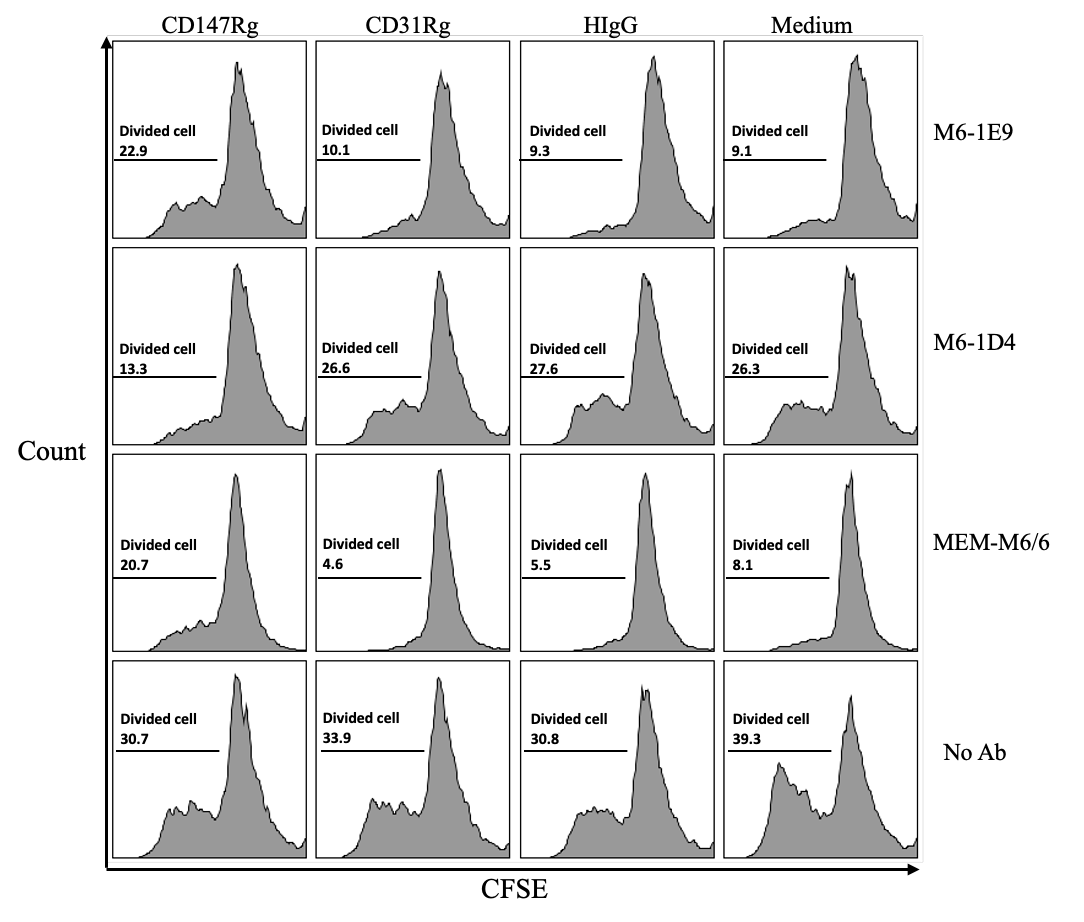
**

**Figure S2. Abolishing of the inhibitory effect of anti-CD147 mAbs by the recombinant CD147 extracellular domain.** PBMCs were stimulated with anti-CD3 mAb (OKT3) in the presence or absence of an anti-CD147 mAb mixture with recombinant CD147 proteins as indicated. The representative flow cytometric data from one of the three individuals were expressed in histogram showing the percentage of divided cell in the indicated conditions.

**Supplementary Figure S3**

**
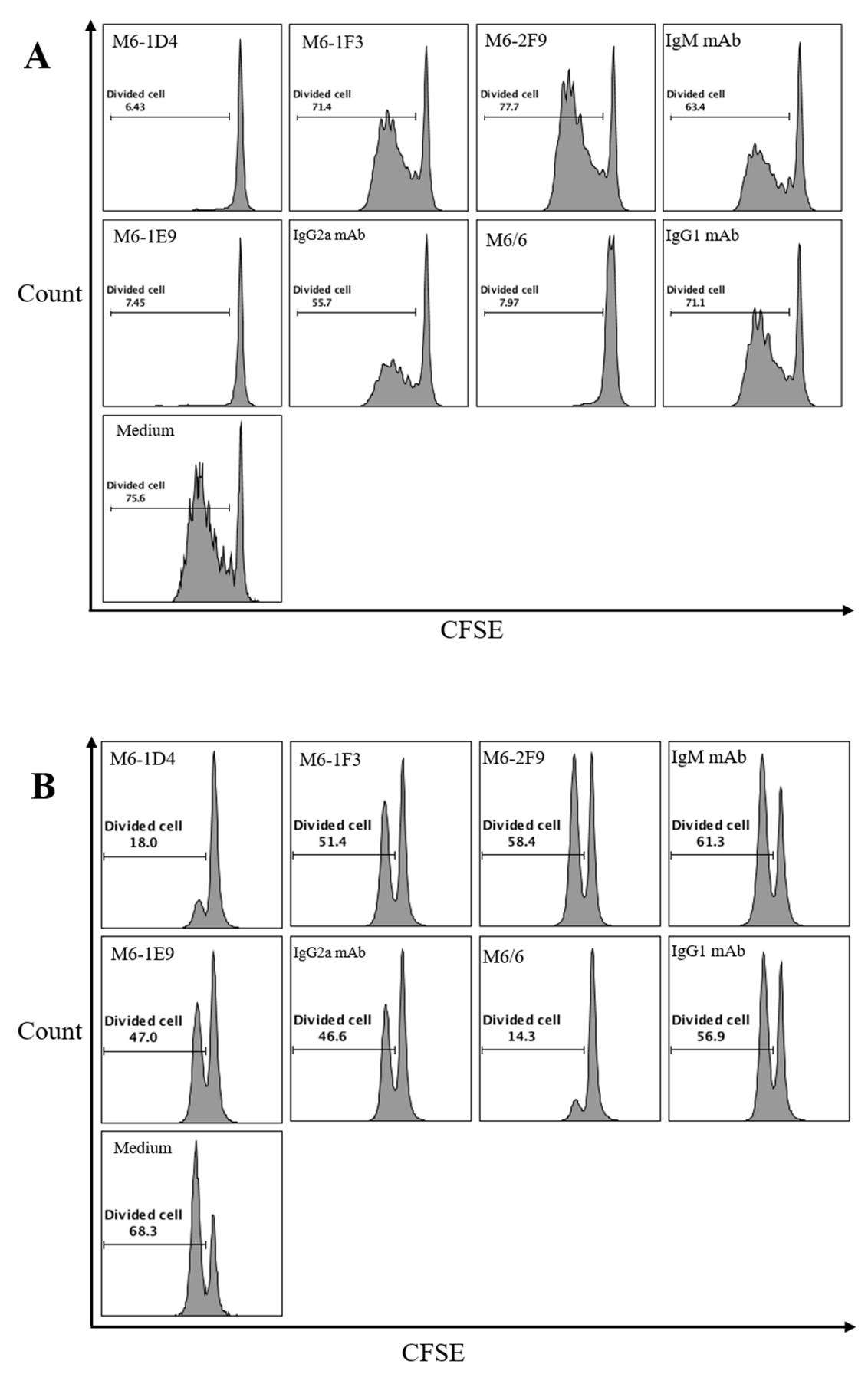
**

**
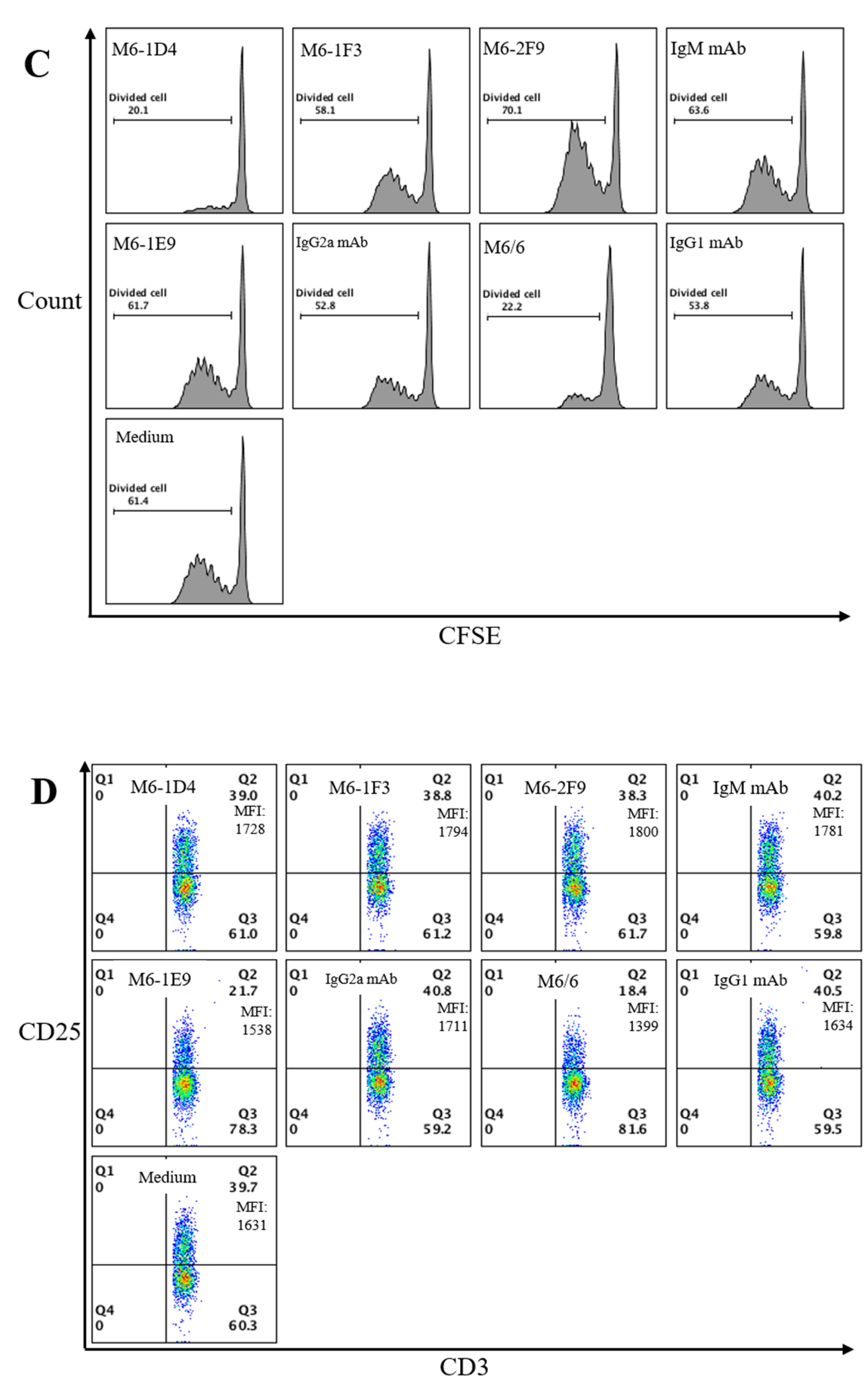
**

**Figure S3. Ligation of CD147 on monocytes and T cells with anti-CD147 mAbs regulates T cell activation.** PBMCs **(A)** and monocyte-depleted PBMCs **(B)** were activated with anti-CD3 mAb. Purified T cells **(C)** were activated with anti-CD3 and CD28 mAbs in the absence or presence of anti-CD147 mAbs or isotype-matched control mAbs. Flow cytometric data were expressed in histograms showing the percentage of divided cells in each condition using CFSE proliferation assay. **(D)** THP1-cells were pre-pulsed with anti-CD147 mAbs or isotype-matched control mAb or medium. The pre-pulsed THP1 cells were co-cultured with PBMCs and activated with anti-CD3 mAb. Flow cytometric data were expressed in dot plot showing the percentage and geometric mean fluorescence intensity (MFI) of the CD25 expressing T cells in the indicated conditions.

**Supplementary Figure S4**

**
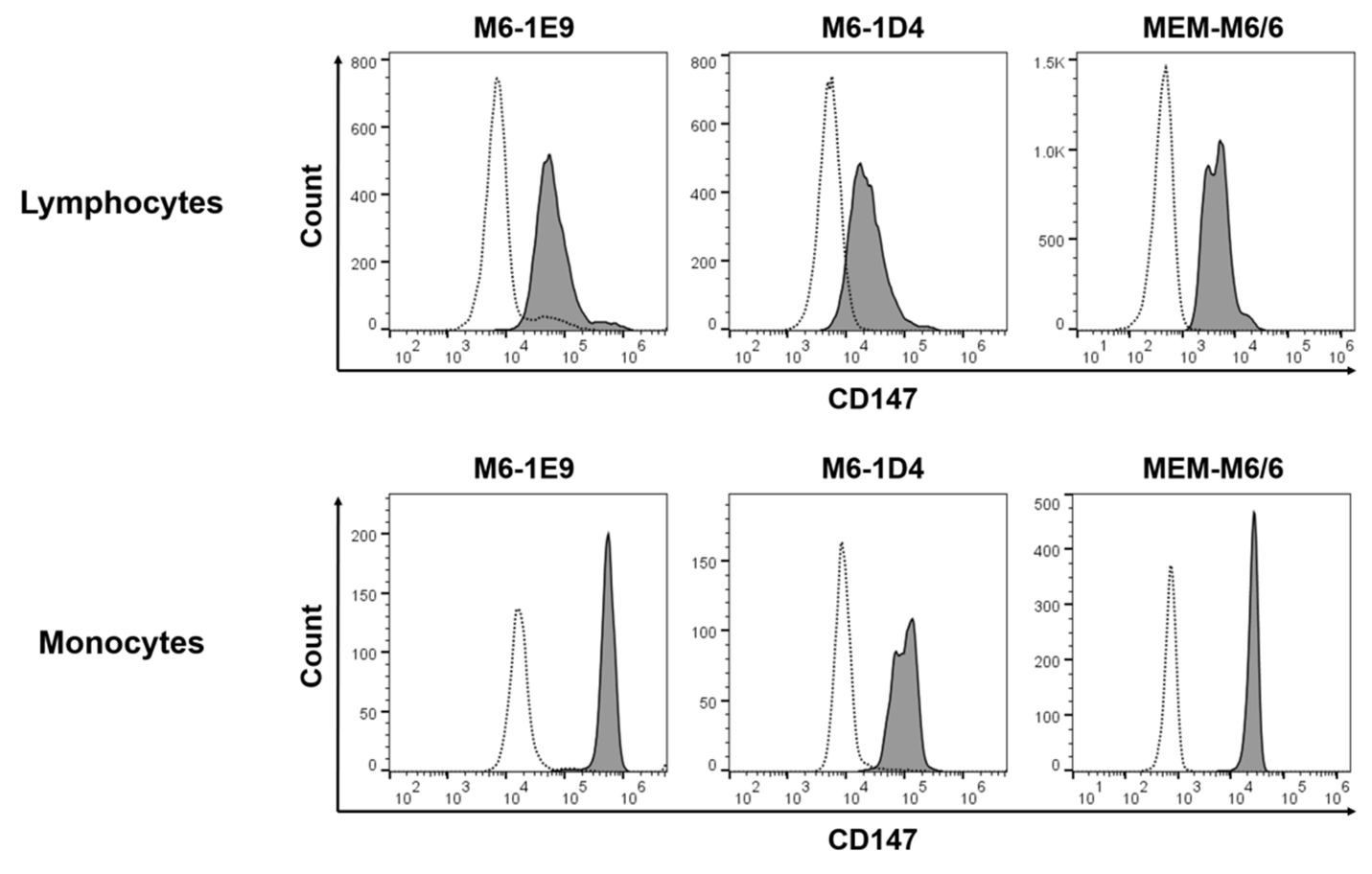
**

**Figure S4. The CD147 expression profiles on PBMCs determined by anti-CD147 mAbs.** PBMCs were stained with anti-CD147 mAbs, M6-1E9, M6-1D4, and MEM-M6/6 as indicated (grey) or isotype-matched control mAbs (white) by indirect immunofluorescence technique. The CD147 expression on lymphocytes and monocytes was analyzed by flow cytometry. The representative flow cytometric data from one of the six individuals were expressed in histogram.

**Supplementary Figure 5**


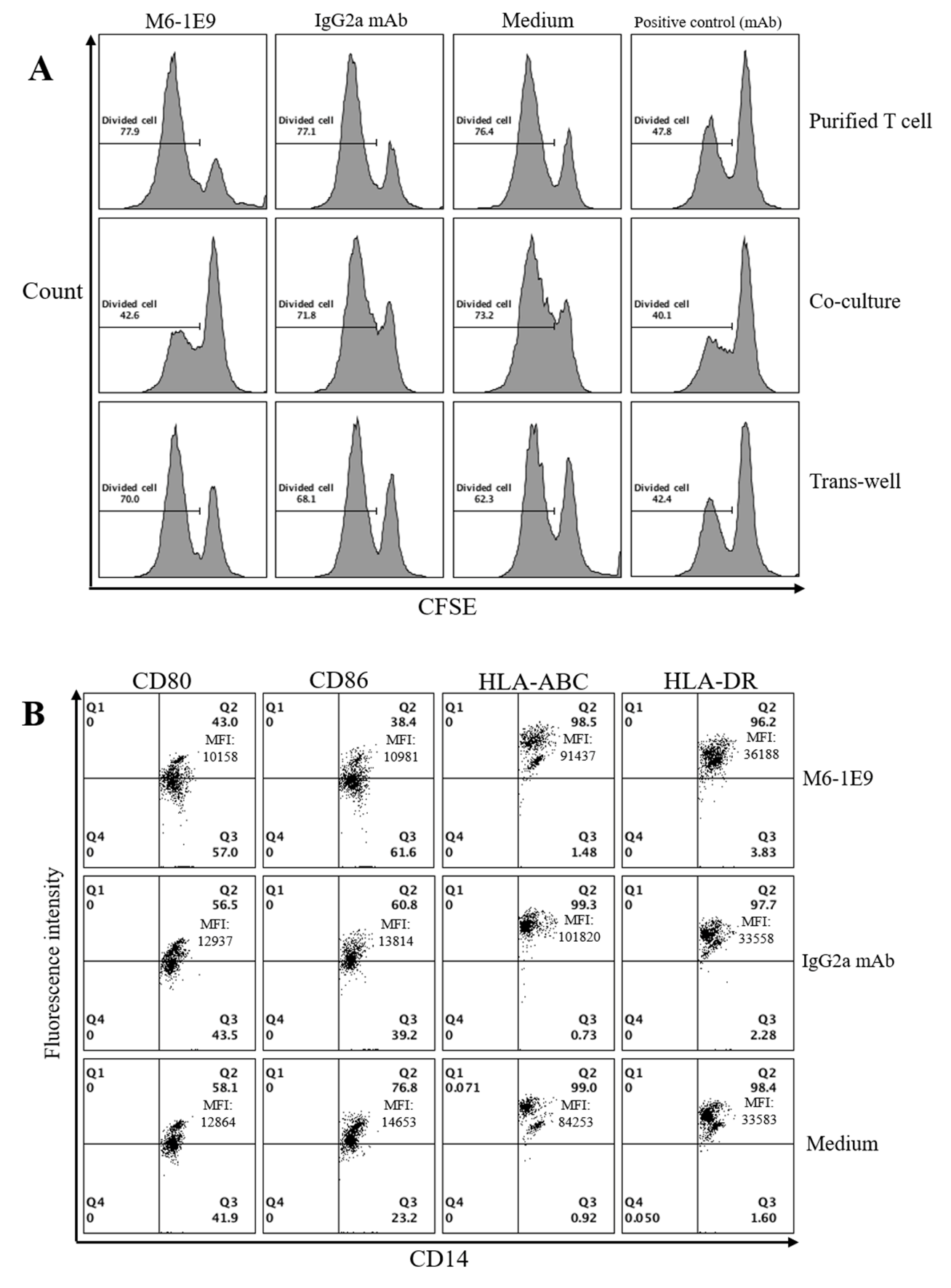


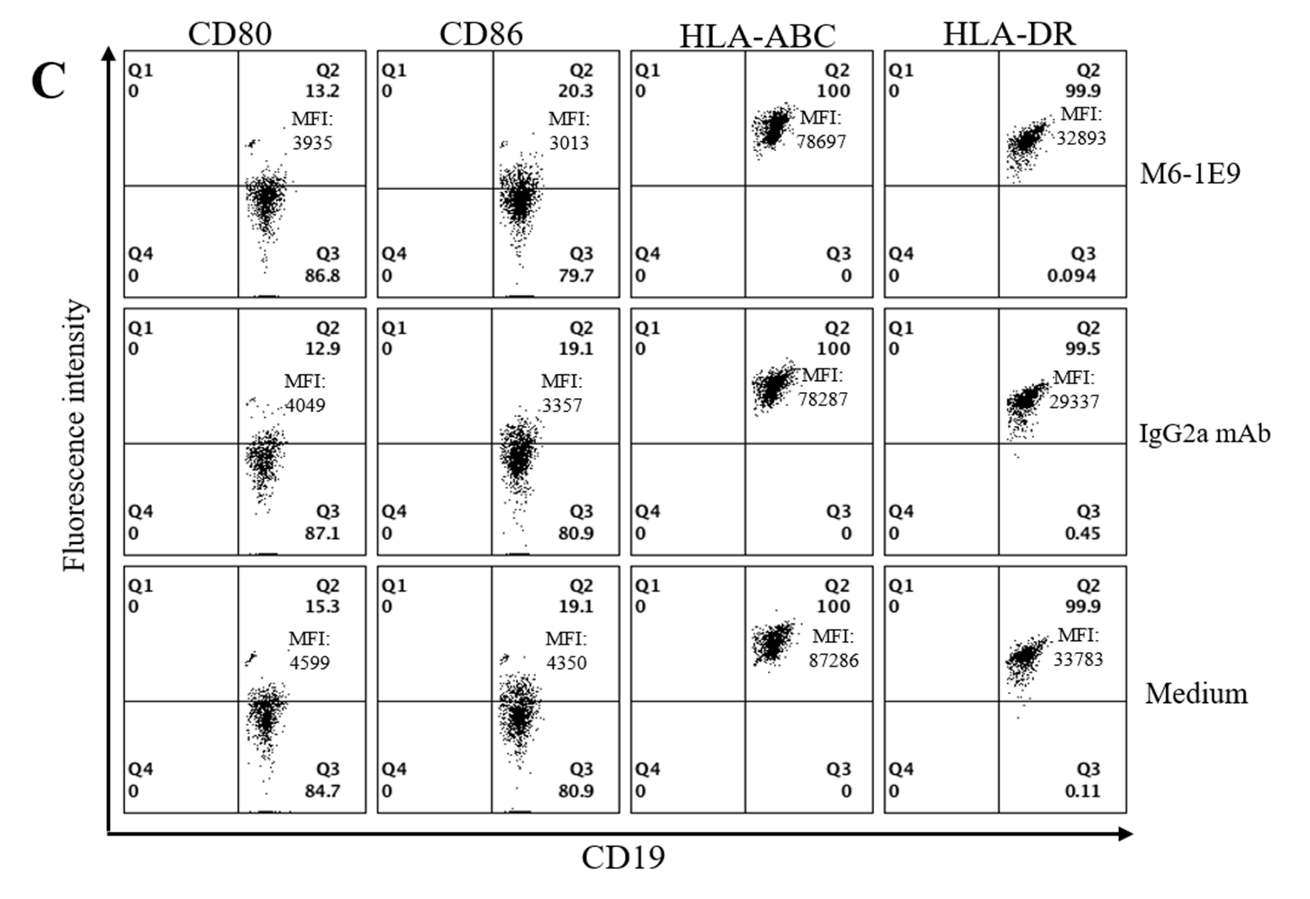


**Figure S5. Ligation of CD147 on monocytes by the anti-CD147 mAb M6-1E9 regulates T cell activation. (A)** Purified T cells and purified T cells cocultured with autologous purified monocytes either in the same well (Co-culture) or in separate compartments (Trans-well) were activated with anti-CD3 mAb in the absence or presence of mAb M6-1E9 or isotype-matched control mAb. The representative histograms show the percentage of divided cells in each condition using CFSE proliferation assay. PBMCs were activated with anti-CD3 mAb in the absence or presence of mAb M6-1E9 or isotype-matched control mAb. The representative flow cytometric data from one of the four individuals was exhibited in dot plot showing the percentage and geometric mean fluorescence intensity (MFI) of the CD80, CD86, HLA-ABC and HLA-DR positive cells on CD14^+^ monocytes **(B)** and CD19^+^ B cells **(C)** in the indicated conditions.
