# Supplementary material for: Differential CD147 Functional Epitopes on Distinct Leukocyte Subsets
Source: Front Immunol. 2021 Aug 4;12:704309. doi: 10.3389/fimmu.2021.704309 (PMC8371324; doi:10.3389/fimmu.2021.704309)
Supplement: Supplementary file 2 [file DataSheet_2.docx]

**
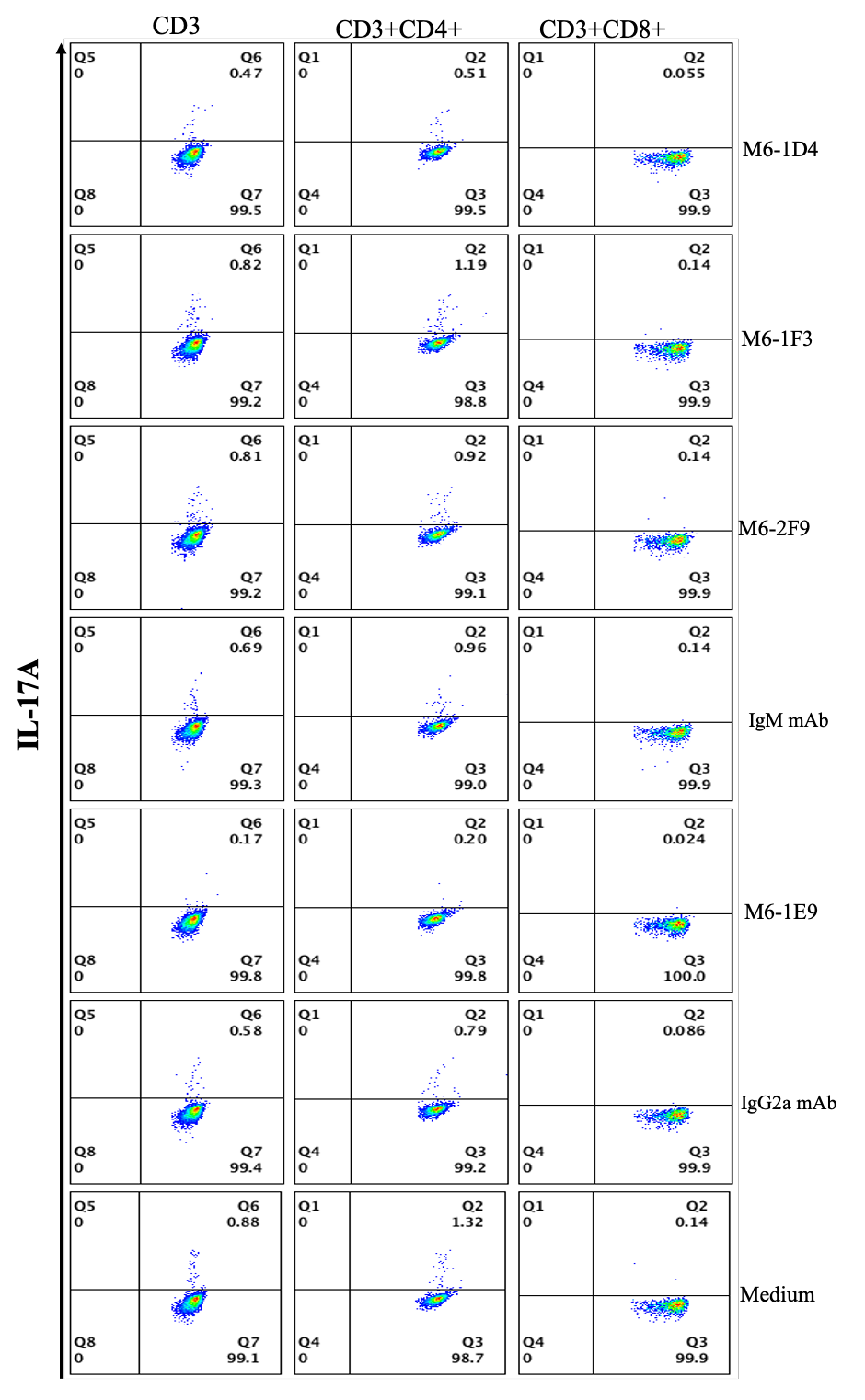
**

**
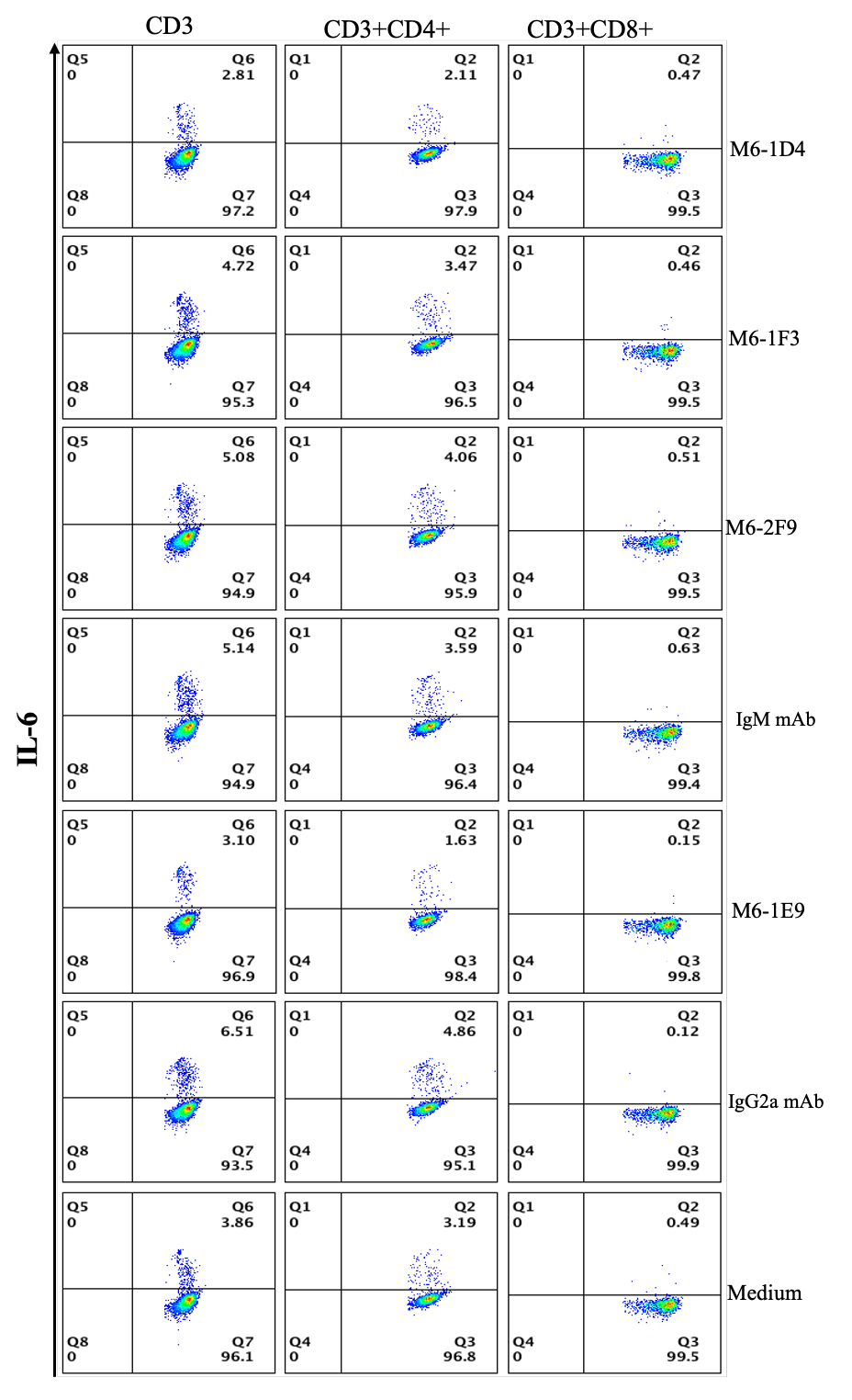
**

**
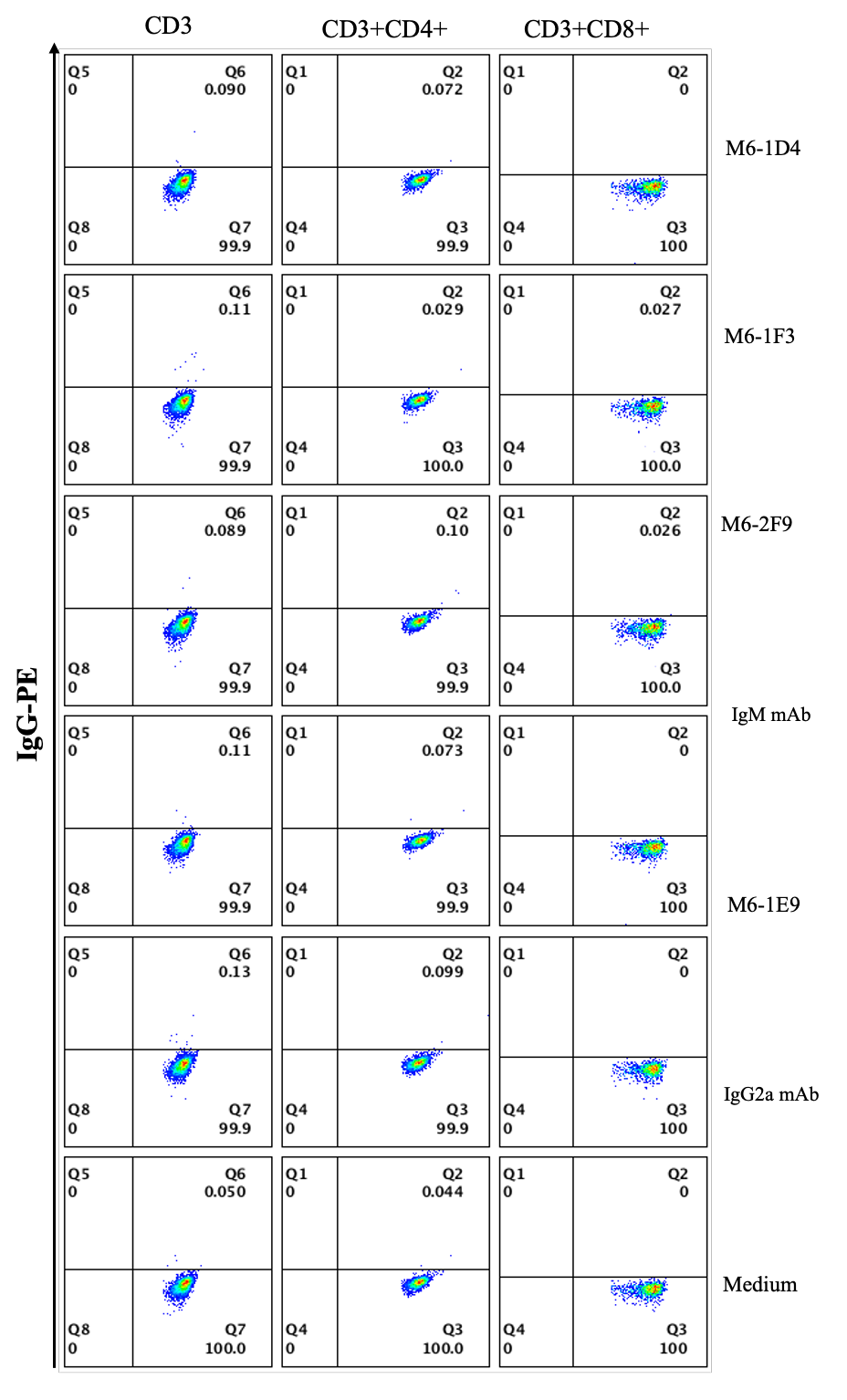
**

**Figure S6. Anti CD147 mAb clones M6-1E9 and M6-1D4 suppressed cytokine production in T lymphocytes.** PBMCs (*n=3*) were stimulated with anti-CD3 mAb in the presence or absence of indicated mAbs. Cells were collected and intracellular stained with antibody against cytokine or control in combination with anti-CD3, CD4, CD8 mAbs. Representative flow cytometric data of CD3+, CD3+CD4+ and CD3+CD8+ cells and indicated cytokine are shown. Percentages of cytokine secreting cells in each treated condition are demonstrated in upper right quadrant.
